# Diagnostic ability of macular ganglion cell asymmetry in Preperimetric Glaucoma

**DOI:** 10.1186/s12886-018-1019-4

**Published:** 2019-01-08

**Authors:** Mei-Ju Chen, Hsin-Yu Yang, Yu-Fan Chang, Chih-Chien Hsu, Yu-Chieh Ko, Catherine Jui-Ling Liu

**Affiliations:** 1 Department of Ophthalmology, Taipei Veterans General Hospital, 201, Section 2, Shih-Pai Road, Taipei, 11217 Taiwan; 2 School of Medicine, National Yang-Ming University, Taipei, Taiwan; 3 Institute of Clinical Medicine, National Yang-Ming University, Taipei, Taiwan

**Keywords:** Macular ganglion cell asymmetry, Optical coherence tomography, Preperimetric glaucoma

## Abstract

**Background:**

To evaluate the diagnostic ability of macular ganglion cell asymmetry to diagnose preperimetric glaucoma (PPG), using Cirrus spectral domain optical coherence tomography (OCT).

**Methods:**

This prospective study included 67 eyes of 67 patients with PPG and 67 eyes of 67 age- and refractive error-matched controls. We measured circumpapillary RNFL (cpRNFL) thickness, macular ganglion cell-inner plexiform layer (GCIPL) thickness and optic nerve head (ONH) parameters using OCT. Macular ganglion cell asymmetries were expressed as absolute difference and ratios between inferior hemisphere and superior hemisphere, inferotemporal (IT) and superotemporal (ST), IT and superonasal (SN), IT and inferonasal (IN), ST and IN as well as temporal and nasal. An asymmetry index was assigned by taking the absolute value of log_10_ of the ratio. The area under the receiver operating characteristics curve (AUROC), partial AUROC (pAUROC) ≥ specificities 90 and 95%, cutoff values and sensitivities at specificities 90 and 95% was analyzed.

**Results:**

Parameters with largest AUROCs were IT GCIPL thickness (0.784), average RNFL thickness (0.767), and average C/D (0.746). For macular asymmetry parameters, log IT/SN index had the largest AUROC (0.734), followed by log IT/IN index (0.725), and absolute difference of IT−SN GCIPL thickness (0.715). Performance was comparable between the best measures of asymmetry analysis (log IT/SN index) and those of cpRNFL, GCIPL, and ONH parameters (all *P* > 0.05). The IT/SN asymmetry index not only had the largest pAUROC based on the pAUROCs ≥90 and 95% specificity (0.044 and 0.019) but also had the highest diagnostic sensitivity at 90 and 95% specificities (52.2 and 46.3%).

**Conclusions:**

GCIPL asymmetry measurements have diagnostic ability comparable to cpRNFL, GCIPL, and ONH analysis for PPG. The best macular ganglion cell asymmetry parameter was IT/SN asymmetry index, which could be a new parameter to detect early structural changes in PPG.

**Electronic supplementary material:**

The online version of this article (10.1186/s12886-018-1019-4) contains supplementary material, which is available to authorized users.

## Background

Glaucoma is characterized by the progressive death of retinal ganglion cells (RGCs) and loss of their axons, with associated visual field (VF) defects. Previous studies indicate that VF defect may not be clinically detectable until 25 to 35% of all RGCs are lost [1–4]. Early detection of structural changes associated with RGCs loss is especially important for preperimetric glaucoma (PPG), which presents with glaucomatous optic disc, retinal nerve fiber layer (RNFL) abnormalities, and normal VF. The introduction of spectral domain optical coherence tomography (OCT) allows for the reproducible and successful segmentation of the inner macular layers. Several studies have shown thinning of the inner retina or RGC complex within the macular area in early glaucoma [5, 6] and PPG [7]. The thickness of macular ganglion cell-inner plexiform layer (GCIPL) may serve as an early indicator of glaucomatous structural damage [8]. OCT has revealed a step-like arcuate defect in the temporal macular GCIPL map in early glaucomatous eyes [9], due to asymmetry in GCIPL thickness distribution between the superior and inferior hemispheres. Using Spectralis posterior pole asymmetry analysis [10–13], Cirrus GCIPL asymmetry [14] or customized software [15, 16], asymmetric glaucomatous macular damage between the inferior and superior hemispheres has been reported in early glaucoma. However, glaucoma frequently starts as a localized thinning of RGC, which might be undetected by averaging values of GCIPL thickness in the designated hemispheric area and its counterpart. Comparison of GCIPL thickness between each parafoveal sector may be necessary to avoid this problem and accurately evaluate ganglion cell asymmetry. In this study, we evaluate the diagnostic ability of the absolute difference, asymmetry ratio, and asymmetry index between six parafoveal macular GCIPL thickness measurements in PPG patients, and compare the asymmetry analysis of GCIPL thickness with traditional cpRNFL, GCIPL, and optic nerve head (ONH) parameters in PPG patients.

## Methods

Patients with PPG who visited the outpatient clinic of Taipei Veterans General Hospital between June 2014 and December 2015 were recruited for this study. We also enrolled age- and refractive error- matched control subjects by recruiting healthy volunteers from the same hospital. The study protocol was approved by the Institutional Review Board of our hospital and was designed in accordance with the Declaration of Helsinki. Written informed consent was obtained from all subjects.

Eyes with focal or diffuse RNFL defects corresponding to glaucomatous optic disc changes and a normal VF test were assigned to the PPG group. Glaucomatous optic disc changes were defined as > 0.7 vertical cup to disc ratio (C/D), > 0.2 asymmetric C/D between the glaucomatous and normal eyes, and neuroretinal rim thinning, notching, or excavation on optic disc photography. Focal or diffuse RNFL defects were based on red-free fundus images. All images including optic nerve head appearance and RNFL defects were evaluated by three glaucoma specialist (M.J.C, Y.C.K and C.J.L.), who were masked to the results of the subjects’ clinical evaluations. A normal VF was defined as a mean deviation (MD) and pattern standard deviation (PSD) within the 95% confidence limit, and a glaucoma hemifield test result within the normal limits by reliable VF test [17]. A reliable VF test was defined as having a fixation loss rate of < 20%, false positive rate of < 33%, and false negative rate of < 33%.

All subjects underwent a comprehensive ophthalmic examination, including assessment of best corrected visual acuity, automated refraction and keratometry, Goldman applanation tonometry, slit-lamp examination, gonioscopy, dilated fundus exam, red-free fundus photography, and automated VF examination (Humphrey 24–2 SITA standard algorithmn). Axial length (AL) was measured using an IOLMaster (Carl Zeiss Meditec, Dublin, California, USA), and central corneal thickness (CCT) was determined using a DGH 55 Pachmate (DGH Technology, Exton, Pennsylvania, USA). Subjects had to meet the following criteria to be enrolled in this study: age ≥ 20 years, best corrected visual acuity ≥20/40, open angle structure upon gonioscopic examination, and astigmatism ≤3 diopters (D). Control subjects were required to have a normal anterior segment on slit-lamp examination, no glaucomatous changes of the ONH and normal VF. Eyes were excluded if they showed retinal or neurologic diseases; ocular inflammation; prior ocular surgery within 3 months; prior refractive surgery, or concurrent disease that could interfere with IOP measurement or OCT imaging or cause VF defects.

**Fig. 1:**
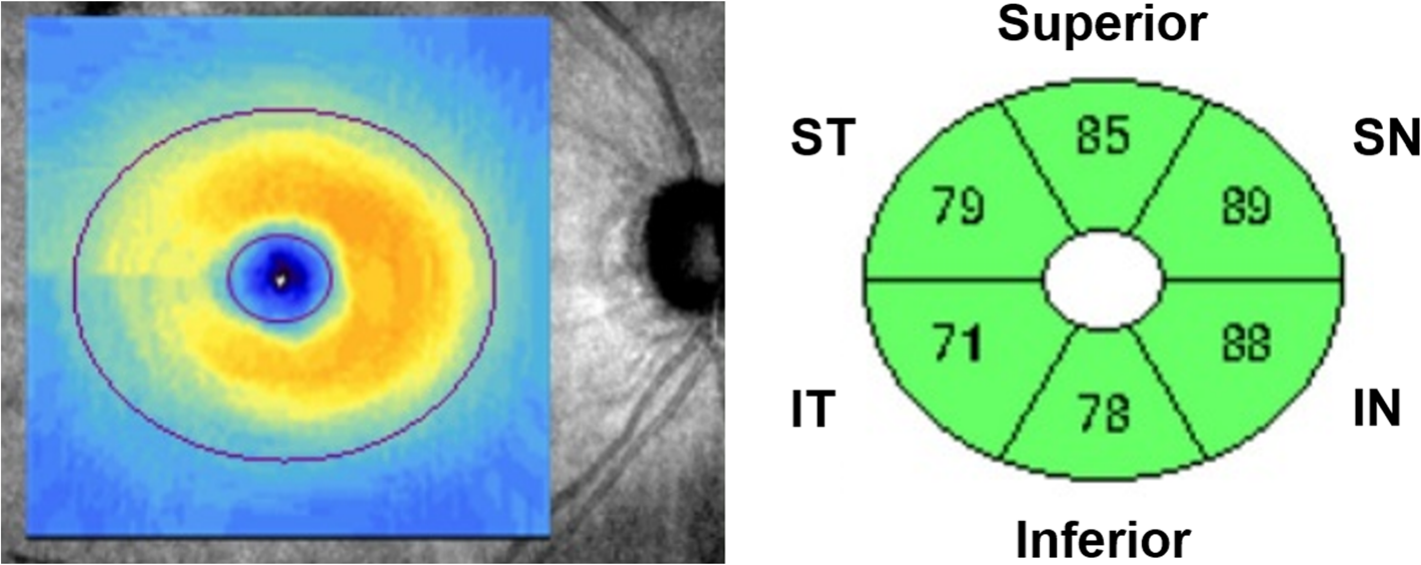
The Cirrus HD-OCT macular cube 200 × 200 protocol provides regional GCIPL thickness in six wedge-shaped sectors

Cirrus HD-OCT (Carl Zeiss Meditec, Dublin, California, USA) was performed following pupillary dilation. The Cirrus HD-OCT Optic Disc Cube 200 × 200 protocol was used to measure ONH rim area, disc area, average C/D, vertical C/D, cup volume, average circumpapillary RNFL (cpRNFL) thickness, and cpRNFL thickness in quadrants and in 12 clock-hour sectors. The Macular Cube 200 × 200 protocol was used to calculate average, minimum, and regional GCIPL thickness in six wedge-shaped sectors (Fig. 1). GCIPL thickness in superior hemisphere (S) was calculated as sum of superonasal (SN), superior and superotemporal (ST). GCIPL thickness in inferior hemisphere (I) was calculated as sum of inferonasal (IN), inferior and inferotemporal (IT). We attempted to compare the difference between I and S, IT and ST, IT and SN, IT and IN, ST and IN as well as temporal (T, sum of ST and IT) vs. nasal (N, sum of SN and IN). In addition to hemispheric comparison, sectorial comparison between six parafoveal sectors were also made for asymmetry evaluation of the GCIPL thickness. Macular ganglion cell asymmetry was expressed as absolute difference (i.e., I–S) and ratio (i.e., I/S) of the thickness between the designated areas. Finally, an asymmetry index was calculated as absolute value of log_10_ of the ratio (i.e., log_10_ I/S). Images were excluded if they exhibited signal strength < 7, motion artifact, poor centration, segmentation error, artifacts caused by ocular pathology, or missing data on the peripapillary region. There was a time interval of < 3 months between HD-OCT and other ophthalmic examination (i.e., VF).

For each subjects, one eye was randomly chosen if both eyes were eligible. The original data was provided as Additional files 1 and 2. Statistical analyses were performed using SPSS version 12.0 (SPSS, Inc., Chicago, IL, USA) and STATA version 12.1 (Stata Corp, College Station, TX, USA). For continuous variables, the normality of data distribution was verified using the Shapiro-Wilk test. We employed Student’s *t* test for normally distributed data, and the Mann-Whitney *U* test for non-normally distributed data to analyze differences between PPG and normal groups. The chi-square test was used to compare the sex ratio. To evaluate the ability of each parameter to discriminate between PPG and normal eyes, we calculated the area under the receiver operating characteristic curve (AUROC), partial AUROC (pAUROC) ≥ specificities 90 and 95%, cutoff values and sensitivities at specificities 90 and 95% for each parameter. The diagnostic performance quantified by AUROC and pAUROC values was compared by using the methods from DeLong et al. [18]. The chi-square test was used to compare the sensitivities at fixed specificities of OCT parameters. For the cpRNFL thickness, GCIPL thickness, GCIPL asymmetry measurements, and ONH parameters comparisons, Bonferroni adjustments were made based on the number of comparisons to correct type I error. For other analyses, *P* value of < 0.05 was considered statistically significant.

## Results

This study included 67 PPG eyes of 67 patients and 67 eyes of 67 age- and refractive error-matched normal controls. The demographic and clinical characteristics of the subjects are summarized in Table 1. There were no significant between-group differences in age, sex, spherical equivalence, AL, CCT, MD, PSD, or visual field index (VFI). However, PPG eyes showed significantly higher intraocular pressure (IOP) compared to controls.

**Table 1:** Demographic and clinical characteristics of the study population

	Normal (*n* = 67)	PPG (*n* = 67)	*P* value
Years of Age	45.3 ± 15.4	48.3 ± 11.1	0.191
Male/Female	29/39	33/35	0.082
SE (D)	−4.25 ± 4.13	−4.62 ± 3.40	0.572
AL (mm)	25.35 ± 1.76	25.60 ± 1.37	0.375
IOP (mmHg)	16.2 ± 3.4	18.4 ± 3.1	0.030
CCT (μm)	557 ± 37	566 ± 36	0.218
MD (dB)	−1.06 ± 1.41	−1.35 ± 1.61	0.265
PSD (dB)	1.83 ± 0.74	1.77 ± 0.58	0.596
VFI (%)	98.9 ± 0.97	98.6 ± 1.61	0.189

*PPG*, preperimetric glaucoma; *SE*, spherical equivalent; *AL*, axial length; *IOP*, intraocular pressure; *CCT*, central corneal thickness; *MD*, mean deviation; *PSD*, pattern standard deviation; *VFI*, visual field index

**Fig. 2:**
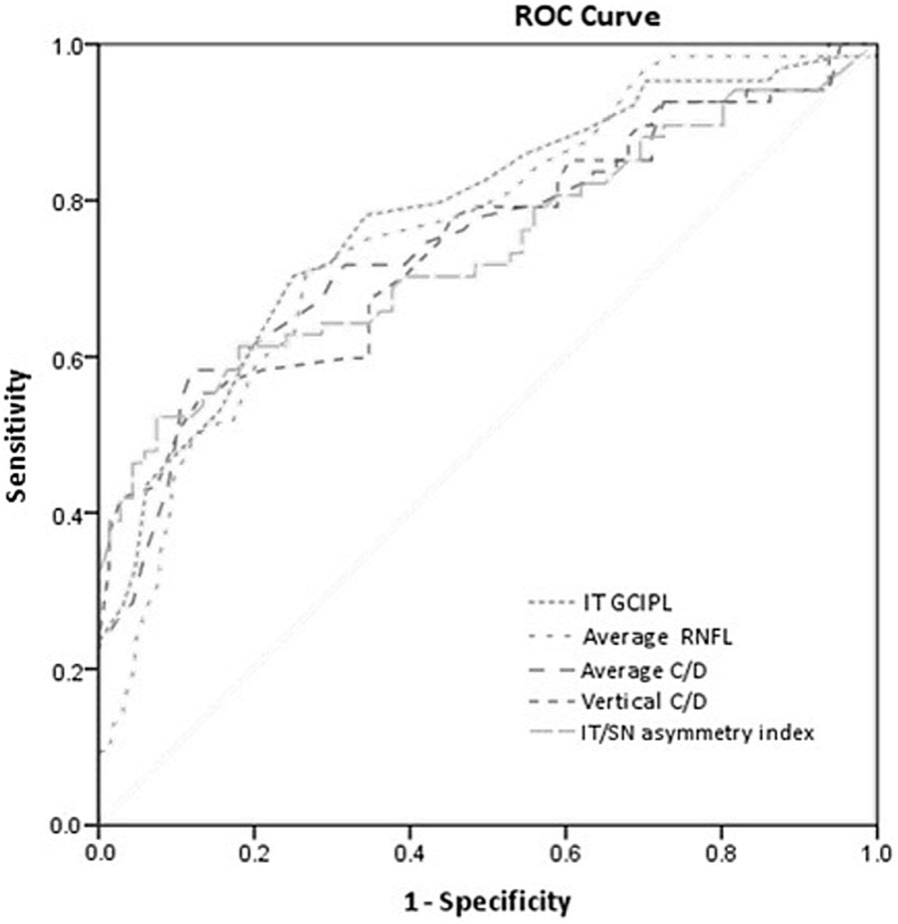
AUROC values for PPG diagnosis. The inferotemporal (IT) GCIPL thickness showed the largest AUROC value (0.784), followed by average retinal nerve fiber layer (RNFL) thickness (0.767), average cup-to-disc ratio (C/D) (0.746), vertical C/D (0.742), and inferotemporal/superonasal (IT/SN) asymmetry index (0.734)

After the Bonferroni adjustment (α = 0. 0014; 36 comparisons), all GCIPL thickness and most cpRNFL thickness (except at the nasal quadrant) measured by HD-OCT were significantly lower in PPG eyes compared to normal eyes (*P* < 0.001) (Table 2). PPG eyes had significantly larger disc area, average C/D, vertical C/D, and cup volume as well as significantly smaller rim area than controls. PPG eyes also had significantly greater GCIPL absolute difference than controls. Significant differences in GCIPL asymmetry ratio were found between the PPG and normal eyes, with the exception of I/S. GCIPL asymmetry index values were significantly different between PPG and normal eyes. Table 3 shows the AUROC and pAUROC values for all the thickness and asymmetry parameters. The IT GCIPL thickness had the largest AUROC value (0.784), followed by average RNFL thickness (0.767), average C/D (0.746), vertical C/D (0.742), and IT/SN asymmetry index (0.734) (Fig. 2). The macular ganglion cell asymmetry parameters with the largest AUROCs were IT/SN asymmetry index (0.734), IT/IN asymmetry index (0.725), and IT–SN GCIPL thickness (0.715). The best individual asymmetry parameters were IT–SN for absolute difference, IT/IN (0.694) for asymmetry ratio, and IT/SN asymmetry index for asymmetry index. Based on the pAUROCs ≥90 and 95% specificity, IT/SN asymmetry index had the largest pAUROC among all parameters (0.044 and 0.019, respectively). Table 4 shows sensitivities at fixed specificities and cutoff values for all OCT parameters. At 90% specificity, IT/SN asymmetry index, average C/D and the cup volume had the highest sensitivity (all 52.2%), followed by vertical C/D and IT–SN (both 50.7%), and IT GCIPL (49.3%). At 95% specificity, IT/SN asymmetry index had the highest diagnostic sensitivity (46.3%), followed by vertical C/D (41.5%) and IT/SN asymmetry ratio **(**40.3%). Table 5 shows the *P* values for pairwise comparison of AUROC values, pAUROC values, and sensitivity at fixed specificities between the best measure of each GCIPL asymmetry analysis and cpRNFL, GCIPL, and ONH parameters. The diagnostic performance was comparable between the best IT/SN asymmetry index and average RNFL thickness (*P* = 0.398), IT GCIPL thickness (*P* = 0.277), and average C/D (*P* = 0.618) based on AUROC values.

**Table 2:** Comparison of cpRNFL thickness, GCIPL thickness and asymmetry measurements, and ONH parameters between two groups

Parametres	Normal (*n* = 67)	PPG (*n* = 67)	*P* value
cpRNFL Thickness			
Average	96.0 ± 9.2	86.8 ± 8.7	< 0.001
Superior	115.1 ± 16.4	104.2 ± 15.3	< 0.001
Nasal	65.1 ± 10.2	64.4 ± 12.2	0.712
Inferior	119.8 ± 17.1	105.9 ± 16.1	< 0.001
Temporal	83.0 ± 14.6	73.7 ± 12.7	< 0.001
GCIPL Thickness			
Average	80.6 ± 5.9	76.2 ± 6.6	< 0.001
Minimum	77.9 ± 6.7	71.8 ± 9.0	< 0.001
Superonasal	82.8 ± 6.7	80.0 ± 9.0	0.043
Superior	81.1 ± 6.6	77.4 ± 7.8	0.004
Superotemporal	80.1 ± 5.4	75.5 ± 7.2	< 0.001
Inferotemporal	80.8 ± 5.6	73.5 ± 7.5	< 0.001
Inferior	77.8 ± 6.6	72.7 ± 7.4	< 0.001
Inferonasal	80.9 ± 7.0	78.0 ± 7.8	0.024
GCIPL Absolute Difference			
I–S	6.1 ± 4.2	12.3 ± 13.6	0.001
IT–ST	2.5 ± 1.7	5.2 ± 5.5	< 0.001
T–N	6.9 ± 4.5	11.9 ± 8.9	< 0.001
IT–SN	3.7 ± 2.4	8.2 ± 7.2	< 0.001
IT–IN	3.3 ± 2.8	6.5 ± 5.6	< 0.001
ST–IN	3.7 ± 3.4	5.0 ± 4.4	0.048
GCIPL Asymmetry Ratio			
I/S	0.98 ± 0.03	0.97 ± 0.08	0.109
IT/ST	1.01 ± 0.04	0.98 ± 0.10	0.019
T/N	0.98 ± 0.05	0.95 ± 0.08	0.002
IT/SN	0.98 ± 0.05	0.93 ± 0.11	0.001
IT/IN	1.00 ± 0.06	0.95 ± 0.09	< 0.001
ST/IN	0.98 ± 0.07	0.97 ± 0.09	0.118
GCIPL Asymmetry Index			
Log_10_ (I/S)	0.01 ± 0.01	0.02 ± 0.03	0.001
Log_10_ (IT/ST)	0.01 ± 0.01	0.03 ± 0.03	< 0.001
Log_10_ (T/N)	0.02 ± 0.01	0.03 ± 0.03	< 0.001
Log_10_ (IT/SN)	0.02 ± 0.01	0.05 ± 0.04	< 0.001
Log_10_ (IT/IN)	0.00 ± 0.02	0.04 ± 0.03	< 0.001
Log_10_ (ST/IN)	0.02 ± 0.02	0.03 ± 0.03	0.026
ONH			
Rim Area	1.21 ± 0.23	1.05 ± 0.19	< 0.001
Disc Area	1.78 ± 0.50	1.98 ± 0.50	< 0.001
Average C/D	0.50 ± 0.20	0.63 ± 0.18	< 0.001
Vertical C/D	0.47 ± 0.20	0.60 ± 0.18	< 0.001
Cup Volume	0.18 ± 0.15	0.36 ± 0.26	< 0.001

*PPG*, preperimetric glaucoma; *GCIPL*, ganglion cell-inner plexiform layer; *cpRNFL*, circumferential peripapillary retinal nerve fiber layer; *ONH*, optic nerve head; *C/D*, cup-to-disc ratio; *I*, inferior hemisphere; *S*, superior hemisphere; *IT*, inferotemporal; *ST*, superotemporal; *T*, temporal; *N*, nasal; *SN*, superonasal; *IN*, inferonasal

**Table 3:** AUROC and pAUROC values for cpRNFL thickness, GCIPL thickness, GCIPL asymmetry measurements, and ONH parameters

Parameters	AUROC (95% CI)	pAUROC at 90% specificity (95% CI)	pAUROC at 95% specificity (95% CI)
cpRNFL Thickness			
Average	0.767 (0.685–0.850)	0.019 (0.004–0.035)	0.007 (0.000–0.013)
Superior	0.697 (0.605–0.789)	0.013 (0.000–0.027)	0.002 (−0.004–0.008)
Nasal	0.523 (0.421–0.622)	0.007 (−0.004–0.014)	0.001 (− 0.001–0.005)
Inferior	0.727 (0.639–0.816)	0.018 (0.002–0.034)	0.005 (−0.003–0.013)
Temporal	0.676 (0.581–0.770)	0.012 (0.001–0.023)	0.005 (0.000–0.009)
GCIPL Thickness			
Average	0.693 (0.604–0.783)	0.014 (0.004–0.023)	0.005 (0.000–0.009)
Minimum	0.720 (0.634–0.807)	0.021 (0.007–0.035)	0.006 (0.000–0.013)
Superonasal	0.632 (0.537–0.727)	0.010 (0.001–0.017)	0.004 (0.001–0.007)
Superior	0.632 (0.537–0.727)	0.010 (0.002–0.018)	0.003 (0.000–0.007)
Superotemporal	0.699 (0.610–0.787)	0.016 (0.003–0.028)	0.006 (0.001–0.011)
Inferotemporal	0.784 (0.707–0.861)	0.035 (0.020–0.050)	0.014 (0.007–0.021)
Inferior	0.695 (0.606–0.784)	0.020 (0.010–0.030)	0.010 (0.005–0.014)
Inferonasal	0.614 (0.519–0.710)	0.007 (−0.001–0.015)	0.002 (−0.001–0.005)
GCIPL Absolute difference			
I–S	0.628 (0.532–0.724)	0.023 (0.013–0.034)	0.011 (0.005–0.016)
IT–ST	0.624 (0.527–0.722)	0.031 (0.019–0.042)	0.014 (0.008–0.020)
T–N	0.661 (0.561–0.754)	0.024 (0.012–0.026)	0.010 (0.004–0.016)
IT–SN	0.715 (0.626–0.803)	0.034 (0.021–0.047)	0.015 (0.009–0.022)
IT–IN	0.704 (0.617–0.790)	0.023 (0.012–0.033)	0.010 (0.004–0.015)
ST–IN	0.600 (0.503–0.697)	0.009 (0.002–0.016)	0.004 (0.000–0.008)
GCIPL Asymmetry Ratio			
I/S	0.604 (0.507–0.701)	0.025 (0.013–0.036)	0.010 (0.005–0.016)
IT/ST	0.606 (0.508–0.703)	0.028 (0.017–0.040)	0.013 (0.007–0.018)
T/N	0.684 (0.592–0.776)	0.033 (0.018–0.047)	0.014 (0.006–0.022)
IT/SN	0.690 (0.598–0.782)	0.039 (0.026–0.052)	0.017 (0.010–0.024)
IT/IN	0.694 (0.603–0.785)	0.031 (0.018–0.043)	0.013 (0.007–0.019)
ST/IN	0.610 (0.513–0.708)	0.013 (0.000–0.026)	0.035 (−0.001–0.009)
GCIPL Asymmetry Index			
Log_10_ (I/S)	0.636 (0.540–0.731)	0.024 (0.012–0.036)	0.010 (0.004–0.016)
Log_10_ (IT/ST)	0.651 (0.556–0.747)	0.036 (0.024–0.048)	0.017 (0.011–0.023)
Log_10_ (T/N)	0.678 (0.585–0.770)	0.026 (0.012–0.041)	0.010 (0.004–0.016)
Log_10_ (IT/SN)	0.734 (0.648–0.820)	0.044 (0.031–0.057)	0.019 (0.012–0.026)
Log_10_ (IT/IN)	0.725 (0.641–0.809)	0.025 (0.013–0.038)	0.011 (0.005–0.017)
Log_10_ (ST/IN)	0.624 (0.529–0.720)	0.010 (0.001–0.019)	0.004 (0.000–0.008)
ONH			
Rim Area	0.716 (0.626–0.806)	0.074 (0.004–0.030)	0.006 (0.000–0.011)
Disc Area	0.636 (0.538–0.734)	0.005 (−0.004–0.014)	0.000 (−0.004–0.004)
Average C/D	0.746 (0.662–0.831)	0.029 (0.013–0.045)	0.012 (0.004–0.019)
Vertical C/D	0.742 (0.656–0.828)	0.028 (0.015–0.041)	0.017 (0.009–0.025)
Cup Volume	0.729 (0.641–0.816)	0.029 (0.009–0.050)	0.007 (−0.004–0.018)

*AUROC*, area under the receiver operating characteristic curves; *pAUROC*, partial area under the receiver operating characteristic curves; confidence interval; *GCIPL*, ganglion cell-inner plexiform layer; *cpRNFL*, circumferential peripapillary retinal nerve fiber layer; *ONH*, optic nerve head; *CI*, confidence interval; *C/D*, cup-to-disc ratio; *I*, inferior hemisphere; *S*, superior hemisphere; *IT*, inferotemporal; *ST*, superotemporal; *T*, temporal; *N*, nasal; *SN*, superonasal; *IN*, inferonasal

**Table 4:** Sensitivities at fixed specificities and cutoff values for cpRNFL thickness, GCIPL thickness, GCIPL asymmetry measurements, and ONH parameters

Parametres	Sensitivity at 90% specificity (%)	Cutoff value*	Sensitivity at 95% specificity (%)	Cutoff value**
cpRNFL Thickness				
Average	41.7	84.5	23.3	80.5
Superior	30.0	93.5	8.3	86.5
Nasal	16.7	53.5	6.7	44.5
Inferior	46.7	100.5	26.7	92.5
Temporal	21.7	64.5	13.3	61.5
GCIPL Thickness				
Average	23.9	72.5	10.4	70.5
Minimum	35.8	70.5	22.3	66.5
Superonasal	11.9	72.5	9.0	70.5
Superior	16.4	72.5	9.0	70.5
Superotemporal	34.3	72.5	20.9	71.5
Inferotemporal	49.3	73.5	34.3	71.5
Inferior	20.9	68.5	19.4	66.5
Inferonasal	13.4	71.5	6.0	66.5
GCIPL Absolute difference				
I–S	31.3	12.5	26.9	13.5
IT–ST	38.8	4.5	32.8	6.5
T–N	29.9	14.5	23.9	15.5
IT–SN	50.7	6.5	35.8	8.5
IT–IN	29.9	7.5	26.9	8.5
ST–IN	10.4	8.5	10.4	10.5
GCIPL Asymmetry Ratio				
I/S	31.3	0.95	28.4	0.95
IT/ST	35.8	0.96	28.4	0.95
T/N	46.3	0.94	35.8	0.92
IT/SN	46.3	0.92	40.3	0.91
IT/IN	37.3	0.93	34.3	0.92
ST/IN	23.9	0.93	16.4	0.91
GCIPL Asymmetry Index				
Log_10_ (I/S)	32.8	0.024	29.9	0.025
Log_10_ (IT/ST)	38.8	0.028	34.3	0.034
Log_10_ (T/N)	37.3	0.037	25.4	0.045
Log_10_ (IT/SN)	52.2	0.036	46.3	0.040
Log_10_ (IT/IN)	29.9	0.042	26.9	0.050
Log_10_ (ST/IN)	11.9	0.049	10.4	0.059
ONH				
Rim Area	28.4	0.945	19.4	0.885
Disc Area	13.4	2.54	3.0	2.84
Average C/D	52.2	0.695	29.9	0.725
Vertical C/D	50.7	0.665	41.8	0.685
Cup Volume	52.2	0.336	29.2	0.467

*AUROC*, area under the receiver operating characteristic curves; *GCIPL*, ganglion cell-inner plexiform layer; *cpRNFL*, circumferential peripapillary retinal nerve fiber layer; *ONH*, optic nerve head; *C/D*, cup-to-disc ratio; *I*, inferior hemisphere; *S*, superior hemisphere; *IT*, inferotemporal; *ST*, superotemporal; *T*, temporal; *N*, nasal; *SN*, superonasal; *IN*, inferonasal

*Based on 90% specificity

**Based on 95% specificity

**Table 5:** *P* values for pairwise comparison of AUROC values, pAUROC values, and sensitivities at fixed specificities between the best measure of each GCIPL asymmetry analysis and cpRNFL, GCIPL, and ONH parameters

	AUROC	pAUROC at 90% specificity	pAUROC at 95% specificity	Sensitivity at 90% specificity	Sensitivity at 95% specificity
IT–SN vs. IT GCIPL thickness	0.277	0.151	0.340	0.474	0.767
IT–SN vs. average cpRNFL thickness	0.398	0.870	0.060	0.304	0.029
IT–SN vs. average C/D	0.618	0.441	0.484	0.667	0.225
IT/IN vs. IT GCIPL thickness	0.141	0.636	0.883	0.250	1.00
IT/IN vs. average cpRNFL thickness	0.247	0.268	0.141	0.553	0.059
IT/IN vs. average C/D	0.414	0.950	0.775	0.386	0.359
Log (IT/SN) vs. IT GCIPL thickness	0.395	0.226	0.290	0.322	0.083
Log (IT/SN) vs. average cpRNFL thickness	0.705	0.017	0.011	0.007	< 0.001
Log (IT/SN) vs. average C/D	0.845	0.145	0.025	0.203	0.008

*AUROC*, area under the receiver operating characteristic curves; *pAUROC,* partial area under the receiver operating characteristic curves; *GCIPL*, ganglion cell-inner plexiform layer; *cpRNFL*, circumferential peripapillary retinal nerve fiber layer; *C/D*, cup-to-disc ratio; *IT*, inferotemporal; *SN*, superonasal; *IN*, inferonasal

## Discussion

In PPG eyes, we find the parameters with largest AUROCs were IT GCIPL thickness, average RNFL thickness, and average C/D. The diagnostic ability of the GCIPL parameters was similar to that of the RNFL and ONH parameters to differentiate from PPG from controls, as Sung et al. [19] and Kim et al. [20] have reported. In contrast, Begum and colleagues [21] show that the diagnostic ability of GCIPL parameters was significantly lower than that of the RNFL and ONH parameters. The discrepancy might be explained by the fact that the axons of RGCs travelling within the RNFL show 100% convergence at the ONH, while the macula area only contain 50% of the total RGCs. OCT measures limited scan area of macular GCIPL, and any RGCs damage outside the elliptical annulus is less likely to be detected by the scan. Furthermore, the standard definition of glaucoma is based primarily on ONH and RNFL changes, rather than macular changes. The bias favoring the ONH and RNFL could underestimate the diagnostic ability of macular parameters [21], even though there is a growing evidence that early glaucomatous damage involves the macula. Using OCT, RGCs damage in the macula is as detectable as the RNFL damage in the classic arcuate regions [22], and the diagnostic ability of GCIPL parameters increased significantly if the RNFL defects are closer to the fovea [20]. The topographic characteristics (angular location and width) of RNFL defects may also affect the performance of OCT. Superotemporal and inferotemporal RNFL bundles tend to converge temporally with increasing myopia [23], so they are more likely to be detected by a macular GCIPL scan. Compared to emmetropic subjects in Begum’s study [21], the PPG patients of Kim et al. [20] as well as in the present study had a mean refractive error of − 1.95 ± 1.56 D and − 4.62 ± 3.40 D, respectively. GCIPL parameters have shown better diagnostic ability than either RNFL or ONH parameters in myopic PPG with a mean refractive error of − 2.92 ± 3.07D [24]. GCIPL maps may have a better ability to detect early glaucomatous damage in the Asian population due to the higher prevalence of myopia compared to other populations.

Among GCIPL parameters, minimum GCIPL thickness had superior diagnostic performance than other parameters for detecting early glaucoma [19, 25]. However, our study showed no diagnostic advantages of minimum GCIPL thickness. This result might be explained by the inclusion of very early glaucomatous patients with minimal structural change. Also, OCT data was compared with the built-in normal database, abnormal thinned macular area defined on color-coded significance map might be misleading (i.e., in myopic eyes). Therefore, measurements of asymmetric GCIPL distribution might be an alternative method in diagnosing PPG, in addition to traditional cpRNFL thickness, GCIPL thickness and ONH parameters. Yamada et al. [26] and Hwang et al. [14] recently reported that inferior-to-superior ganglion cell asymmetry index had good diagnostic ability in PPG and early glaucoma. Similarly, diagnostic performance of the Spectralis macular hemifield asymmetry was comparable to sectoral cpRNFL thickness in early glaucoma [11, 12]. However, GCIPL asymmetry could be underestimated in a simple comparison between the averaged values of two or three sectors of GCIPL thickness, such as the inferior hemisphere versus the superior hemisphere. Compared to previous “hemispheric-based” studies, “sector-based” asymmetry analysis was expressed as difference between each six parafoveal sectors in the present study. We found the IT/SN GCIPL asymmetry index best discriminated PPG from controls based on either pAUROC or diagnostic sensitivity at 90 and 95% specificity. To our knowledge, this is the first study to demonstrate this new diagnostic parameter for PPG. Topographically, GCIPL thinning occurred in the inferior and temporal portions of the macula in early glaucoma [27], and the IT GCIPL has shown to be the most sensitive sector [24]. In the macular area, the peak density of RGCs occurs at paracentral 3.7 degrees and is thicker in the superior portion than in the inferior portion of the macula [28]. Histological studies also show that the temporal and inferior sectors have fewer ganglion cells than the superior and nasal sectors [29]. The superiority of the IT/SN asymmetry index might be explained by the anatomic changes between inferotemporal sector and its counterpart (superonasal sector). Therefore, IT/SN asymmetry index appears to be a valuable parameter in diagnosing PPG.

These results show that the diagnostic ability of GCIPL asymmetry measurements was comparable to that of GCIPL thickness analysis for PPG, consistent with a previous asymmetry study [14]. Another study showed that GCIPL asymmetry had inferior diagnostic performance than that of the GCIPL thickness parameters [30], which might be due to the differences between absolute difference, ratio, and calculated index. The absolute difference directly reflects the amount of change, while the ratio indicates the relative amount of change considering the baseline level [14]. The asymmetry index further amplifies the relative amount of change by using a logarithmic calculation. It is less dependent on the severity of glaucoma than were thickness measurements. Particularly in the pre-perimetric stage, the amount of glaucomatous change is relatively small compared to the changes seen in other stages of glaucoma. Although the localized thinning of the ganglion cell layer might not affect the average thickness, it might substantially affect the asymmetry index. Therefore, the Log_10_ asymmetry index appears to have an increased ability to detect glaucoma than simple difference and ratio, and could be a new indicator of early localized glaucomatous damage.

This study had several limitations. First, the sample size was relatively small. A future study of larger sample size should be conducted. Second, all of the study subjects were of Chinese ethnicity and therefore the results cannot necessarily be extrapolated to patients of other ethnicities. Third, the definition of PPG was for this study. The enrolled criteria could not ensure that all participants are PPG, only prospective follow-up could provide enough evidence for the diagnosis. No distinct evidence of progression could be observed to differentiate true PPG from glaucoma suspect because this is a cross-sectional observation study. Forth, we recruited PPG based on glaucomatous structural change and normal VF findings, which is defined as normal hemifield test that is symmetric around the horizontal meridian. This could exclude glaucomatous eyes with very early symmetric function change. This means that the diagnostic ability of asymmetry parameters may be underestimated compared with that of thickness parameters. On the contrary, we might have recruited a majority of eyes with RNFL defects more close to fovea or damage in the inferior macula, which may overestimate the diagnostic ability of IT/SN asymmetry index. Lastly, the influence of test-retest variability on the asymmetry analysis was not considered. The performance of the built-in software algorithm of HD-OCT may affect the results. Despite these limitations, the present results are relevant to discriminating between PPG and normal eyes in clinical practice. In addition, the asymmetry measurements don’t require any special software and can be easily calculated, and the GCIPL asymmetry parameters are helpful for early detection of glaucoma.

## Conclusions

In the present study, we used to spectral domain OCT to evaluate the diagnostic ability of the absolute difference, asymmetry ratio, and Log_10_ asymmetry index between six parafoveal macular GCIPL thickness measurements in PPG patients, and compare the asymmetry analysis of GCIPL thickness with traditional cpRNFL, GCIPL, and ONH parameters in PPG patients. Our results showed the diagnostic ability of GCIPL asymmetry measurements was comparable to that of cpRNFL, GCIPL, and ONH analysis for eyes with PPG. The IT/SN asymmetry index performed best of all the asymmetry analyses and may serve as a new parameter for detecting early structural changes in PPG.

## Additional files

Additional file 1: The original data of normal group. (XLSX 20 kb)

Additional file 2: The original data of PPG group. (XLSX 21 kb)

## Abbreviation

AL: axial length
AUROC: area under the receiver operating characteristic curves
C/D, VF: visual field; cup-to-disc ratio
CCT: central corneal thickness
CI: confidence interval
cpRNFL: circumferential peripapillary retinal nerve fiber layer
D: diopters
GCIPL: ganglion cell-inner plexiform layer
I: inferior hemisphere
IN: inferonasal
IOP: intraocular pressure
IT: inferotemporal
MD: mean deviation
N: nasal
OCT: optical coherence tomography
ONH: optic nerve head
pAUROC: partial area under the receiver operating characteristic curves
PPG: Preperimetric glaucoma
PSD: pattern standard deviation
RGCs: retinal ganglion cells
S: superior hemisphere
SE: spherical equivalence
SN: superonasal
ST: superotemporal
T: temporal
VFI: visual field index
